# The impact of lecture playback speeds on concentration and memory

**DOI:** 10.1186/s12909-023-04491-y

**Published:** 2023-07-18

**Authors:** Zachary I. Merhavy, Lukas Bassett, Michelle Melchiorre, Maureen P. M. Hall

**Affiliations:** Ross University School of Medicine, Bridgetown, Barbados

**Keywords:** MedicalEducation, PlaybackSpeed, Concentration, Retention, Lecture, LongTermMemory

## Abstract

**Background:**

The information required to be learned and retained by medical students has continued to increase over the years. The stress that medical students face has already been highlighted in several studies, however, this in combination with a post-pandemic educational system subsequently generated a shift in medical education towards asynchronous streaming of daily lectures as part of the curriculum with variable playback speed options.

**Methods:**

This paper aims to study the effectiveness of playback speeds, principally that of 1.5x and 2x playback speeds. One objective of this study is to analyze the existing literature regarding how playback speeds may impact learning, and to highlight the need for additional research. It has become apparent that there is not enough literature to support the role that playback speeds have in concentration and/or long-term memory retention in medical students. Due to this lack of information on the topic, this paper additionally highlights a study conducted on second year medical students at one university to assess the associations that may exist between lecture playback speeds of 1.5x and 2x and concentration and long-term retention of memory.

**Results:**

Based on the data collected, it was found that there was no significant difference in student concentration or long-term memory retention with regards to lecture playback speeds.

**Conclusions:**

Although more studies are needed to better understand the topic, it is the current recommendation of the authorial team that students are free to watch medical school lectures at their preferred speed without worry of changes in learning ability.

## Background

The information required to be learned and retained by medical students has continued to increase in conjunction with advancements in the field of medicine. Consequently, students are expected to learn a higher volume of information within their medical curriculum, consisting of didactic lectures that are synchronously streamed and unrecorded. This imposes more burden on the alignment of time and workload in the daily life of a medical student. Therefore, digital audio media in medical education is a natural shift that allows asynchronous daily lectures streaming and is an integral part of the curriculum. An illustration of audio technology is recorded lectures with variable playback speeds, which enable students to watch an hour-long lecture in as little as 30 to 45 min.

A recent study by Hill et al. in 2018 administered an open-ended questionnaire to medical students. The results showed that the most significant stressors of the study were time constraints, lack of balance, and workload, which can negatively impact medical students’ health and wellness [1]. This notion was previously illustrated in a 2012 study that surveyed 562 medical students between 1st and 3rd year, finding depressive symptoms reported by 60% of participants [2]. Another study compared medical students with humanities students, discovering a four-to-five-fold increase in rates of anxiety and depression in medical students as compared to humanities students [3]. Given these observations, exploring the application and effectiveness of variable playback speed on memory and concentration is worthwhile.

Using Google Scholar, keywords “video playback,” “education,“ “sped up,“ “comprehension”, and “medical” were sequentially added to the search retrieving up to 20 results. The term “2x” was to be included. However, no results matched this query when applied to the search sequence. Of the 20 results, one study of 54 medical students displayed a significant finding that watching a lecture at faster speeds may have an adverse or no significant effect on learning new content [4]. There were only three citations to the article, and none related to playback speed. Thus, a second search was performed in the same manner as the first using “time compression,” “multimedia,” “education,” “retention,” “performance,” “learning,” “audio,” “visual,” “comprehension,” “academic,” “recall,” “focus,” and “sped up” until five results remained. One study from this search showed a primary finding that revealed no significant difference in memory retention between the treatment and control group regarding time-compressed multimedia instruction [5]. This study referenced two authors, Pastore and Ritzhaput, nine times. Therefore, additional articles were found through their associations with Pastore and Ritzhaput and Google Scholar searches.

One of the earliest studies investigating playback speed and retention is from 1968, using tape recorders [6]. This study of 118 psychology students at the University of Buffalo investigated the effects of a 1.5x playback speed increase on immediate recall and recall after two weeks [6]. The control lecture was 21 min long, with the experimental groups being 14 min long [6]. Retention was measured using a 30-question test, where the results displayed that an increase in word rate by one-third showed no significant loss in retention [6].

Similarly, in a randomized trial of 54 medical students at the University of Kentucky, Song et al. investigated lecture playback at different speeds [4]. This study compared 1x and 1.5x playback speeds of 2 various recorded lectures, where one was 12 min and the other 15 min in length [4]. Students were divided into either group A or group B, where if group A watched the 12-minute lecture at 1.5x speed, they would then watch the 15-minute lecture at normal speed and serve as the control, and vice versa for group B [4]. Retention was then measured using a 20-question post-test [4]. The results concluded that 1.5x playback speed showed either no effect or a detrimental effect on immediate recall when learning novel information, which supported the notion that faster playback speeds may jeopardize the memory retention of learners [4].

Another study compared three different playback speeds to analyze the relationship between playback speed and verbal redundancy [7]. One hundred and eighty-three graduate students from North Carolina Wilmington University participated, where three groups watched a video about podcasting in education at one of three audio speeds; 1 × (150 words per minute [wpm]), 1.4 × (210 wpm), and 1.8 × (270 wpm) [7]. Respectively, the video lengths (minutes: seconds) were 20:54, 14:59, and 11:40, and the videos were watched with and without verbal redundancy [7]. Using a 10-question post-test, no significant difference in performance between the playback speeds was found [7]. A satisfaction survey also found that the participants preferred 1.4x playback speed and considered it more enjoyable [7].

While memory retention of copious amounts of information remains a necessity for students pursuing a career in medicine and given the time constraints in which they must do so, digital audio media with variable playback speeds has been offered as a solution. However, there is little to no research relating to the effects of increased playback speeds and memory retention in medical education, or academics in general, for that matter. Based on the above literature review, only one article [4] involved medical students yet had contradictory results to both Barabasz and Ritzhaupt’s studies [6, 7]. Moreover, there has been no consistency or attempt at reconstruction in the research methods. Barabasz’s study was purely audio and has been the only study thus far to analyze long-term retention [6]. Ritzhaupt’s investigation found no difference between 1x, 1.4x, and 1.8x playback speed concerning performance; however, the study focused on analyzing the effects of verbal redundancy on compressed audio [7]. Additionally, Song’s study found that 1.5x playback speed had no benefit or a deleterious effect on performance [4]. One key factor is that for all the studies mentioned, none of the videos were over 21 min, whereas, at a typical medical institution, the average medical school lecture presentations are usually 45 min or greater [8, 9].

Medical students are already strained with the information they are required to learn, as illustrated in Hill et al. [1–3]. To mitigate this, the authors believe students utilize the increased playback speeds to cover more information in less time. However, the efficacy of video playback speed on learning has not been fully elucidated, nor has a consensus been identified from the literature reviewed. It is, therefore, important to gain insight into whether this is a viable strategy for students. The researchers believe that viewing lectures at increased playback speeds results in diminished memory retention and more significant concentration fatigue than watching lectures at the standard speed.

## Methods

### Recruitment, consent, survey instruments, and participant inclusion & exclusion

Upon obtaining approval from the Ross University School of Medicine (RUSM) Institutional Review Board, research participants were recruited via email and sent to all students in their 5th and final semester of medical sciences at RUSM. The email described the study opportunity and contained details of an informed consent linked to SurveyMonkey®, where they would complete a survey asking which playback speed they used and how often it was used. The survey link first took each participant to a page containing the consent details, including contact information, in the event of the need to address the question(s) or concern(s) about the research study. Participants were instructed to read the consent and then complete a survey that implied acceptance of the consent terms. They were informed that submitting the survey indicated that they had read and understood the consent and, thus, met the inclusion criteria as research participants. If participants disagreed with the consent, they were advised not to complete the survey and excluded from the research study. Students were also ineligible if they did not complete all the assigned tasks detailed in the subsequent sections of the survey instruments.

### Video selection, interface, and instructional guidelines

The lectures were selected from the content one week before the scheduled delivery in the curriculum. Thus, student research participants had the opportunity to engage in the content using the instructional guideline given during the research event before the official lecture presentation. The authors chose to use students’ medical school lectures not only as an additional incentive but also to address the use of playback speed in medical education accurately.

In addition, the authors decided to use a pharmacology lecture on pulmonary drugs for COPD (chronic obstructive pulmonary disease) and asthma. One reason for selecting this lecture was that the lecturer spoke at a neutral and consistent pace. The second reason for this lecture selection was that it was a lecture on a more straightforward topic. This meant it only required a little additional conceptualization as, for example, a pathology lecture on heart failure a patient may have.

The medical lecture format was consistent in how lectures are presented at the authors’ institution; the audio and side image of the lecturer speaking, with images of the slides as the lecturers went through them. Participants would watch a lecture at either 1.5x or 2x playback speed. The standard length of a lecture at our institution is approximately 50 min. Therefore, participants would finish their lectures in 33 or 25 min. Both before and after the lecture, the participants would complete a pre- and post-lecture quiz on the topic and a pre- and post-lecture concentration game.

Participants would initially join a Zoom® meeting where the investigators went over the itinerary of activities for the study. Student-research participants were explicitly told not to look up answers during the quizzes and neither to take notes nor look at additional study materials during the study. Following the briefing, participants were randomly assigned to a 1.5x playback or 2x playback group using the Zoom® breakout rooms feature. The authors chose to use 1.5x speed as the control group due to the study by Song et al., which found no difference between those listening to 1x versus 1.5 playback speed [4]. Additionally, if this study were to have a third group, it would have reduced the number of participants per group if turnout were low.

### Data collection

**Fig. 1:**
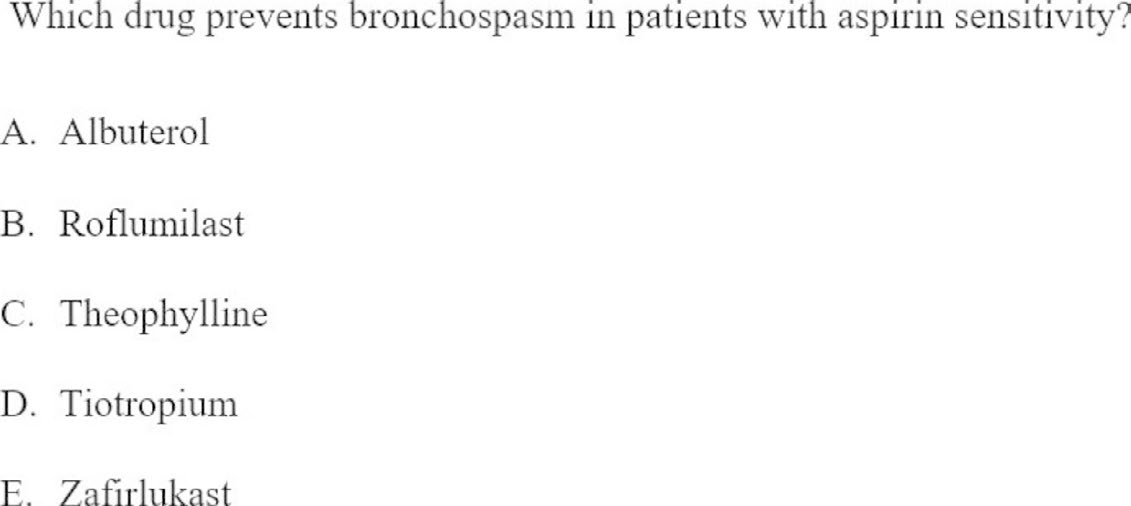
Example of a 1st order question used in both the pre-and post-quizzes

**Fig. 2:**
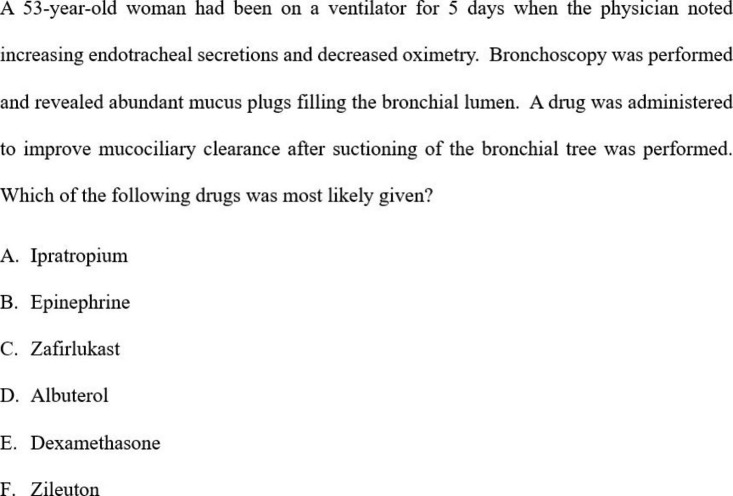
Example of a 2nd order question used in both pre- and post-quizzes

The participants accessed the pre- and post-lecture quiz on the RUSM student portal and had 90 s to complete each question, and the participants were unaware of how they performed on the test. The questions used were 1st and 2nd order multiple choice questions consisting of four to five choices, written by the professor who delivered the lecture. Figures 1 and 2 are examples of quiz questions asked in pre- and post-quiz.

The authors initially had a 5-question quiz but increased the quiz to 10 questions for two subsequent cohorts. The pre- and post-lecture game consisted of a grid with matching images only visible when clicked. The participant would then have to remember where the other matching picture was located on the grid. If the selection were wrong, both images would flip down; if correct, both would stay up. The authors intended to compare pre- and post-lecture game scores to measure memory and the time in minutes to complete the game as a measure of concentration. The pairing of the quizzes with a concentration game was intended to measure participants’ concentration levels after completing a task. As students watch lectures with higher playback speeds to accomplish more in a shorter time, they are presumed to be moving to another lecture or assignment after watching a lecture. However, the measurement was changed from time to completion in the first cohort to the total number of attempts in the second and third cohorts of participants. This was done to mitigate the rapid clicking of sections on the grid. The games used respectively were “Concentration” (Play.vg) and “Memory Game” (MemoryGame.org).

### Analysis

The results from the pre- and post-lecture games were associated with the individual student accounts, so a qualified faculty member collected the results and then provided them to the authors. This data was then de-identified and compiled into an Excel document for analysis. The authors concluded that an unpaired t-test would be an adequate data collection and presentation method. After project approval by the RUSM institutional research committee, it was agreed upon that an unpaired t-test would be appropriate.

## Results

This study was conducted to determine the effect of watching medical school lectures at faster than 1x playback speeds on medical students’ concentration and memory retention. Within the study, three (3) cohorts were assessed by watching a standardized lecture at both 1.5x speed and 2x speed. All cohorts were given a pre-concentration game, a pre-lecture quiz, a post-concentration game, and a post-lecture quiz to measure the students’ concentration and memory retention.

**Fig. 3:**
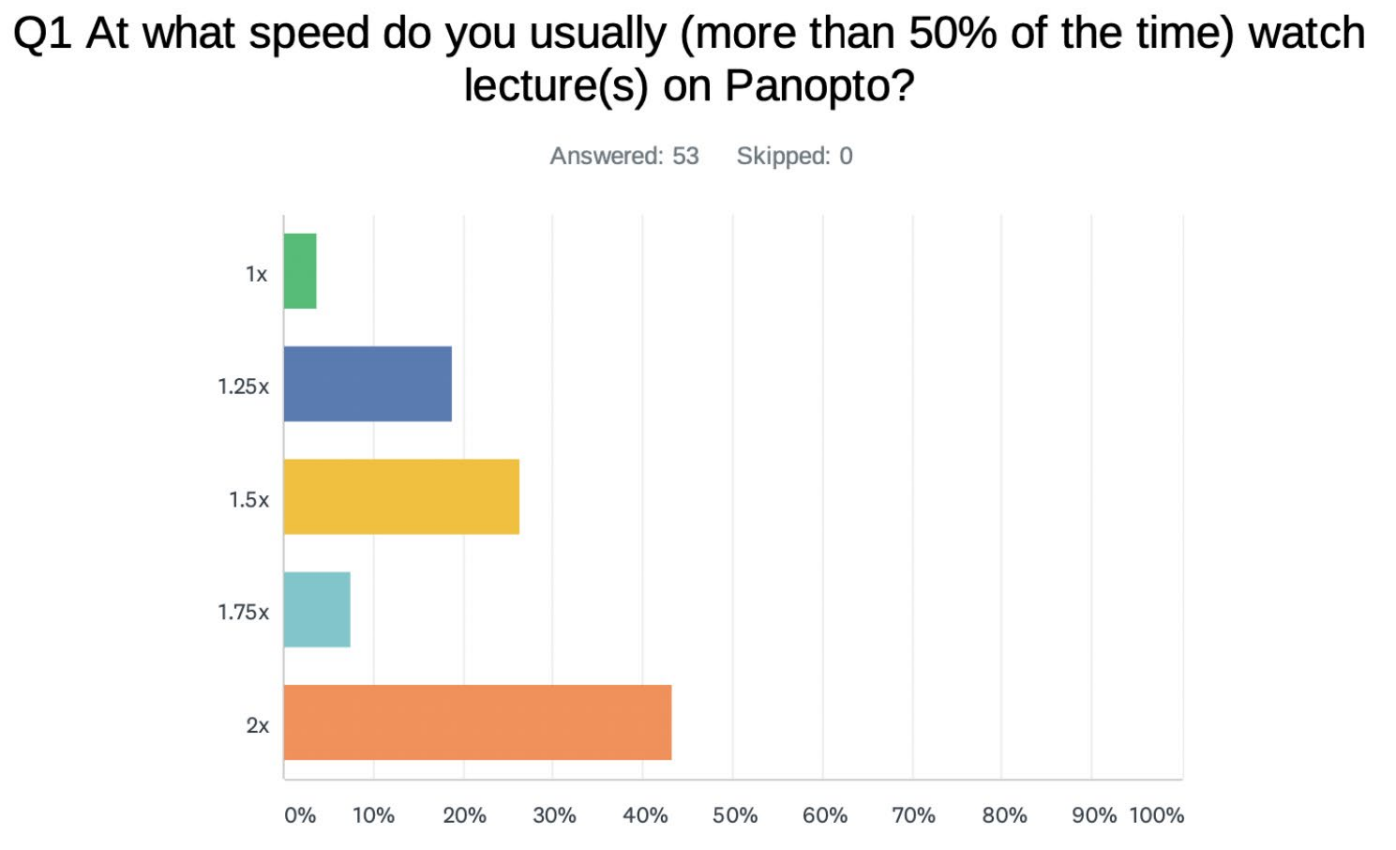
Average speed of lecture viewing by selected students

Before the start of the study, fifty-three (53) of the sixty-six (66) participants completed a pre-study survey about their current study habits. Cohorts 1, 2, and 3 comprised 31, 14, and 21 students, respectively. One question specifically sought to compare students’ current lecture playback speeds, as seen in Fig. 3. Of the 53 participants who completed the survey, 2 (3.77%) watched lectures at the standard 1x speed. Ten participants (18.87%) typically watch lectures at 1.25x speed, and 4 (7.55%) watch lectures at 1.75x speed. Fourteen participants (26.42%) typically watch lectures at 1.5x speed, whereas 23 participants (43.40%) typically watch lectures at 2x speed. Panopto® is an online lecture recording and playback software that allows individuals to watch a camera-recorded lecture and the real-time screen recording concurrently, allowing users to watch at speeds ranging from 0.5x-2x speed.

**Fig. 4:**
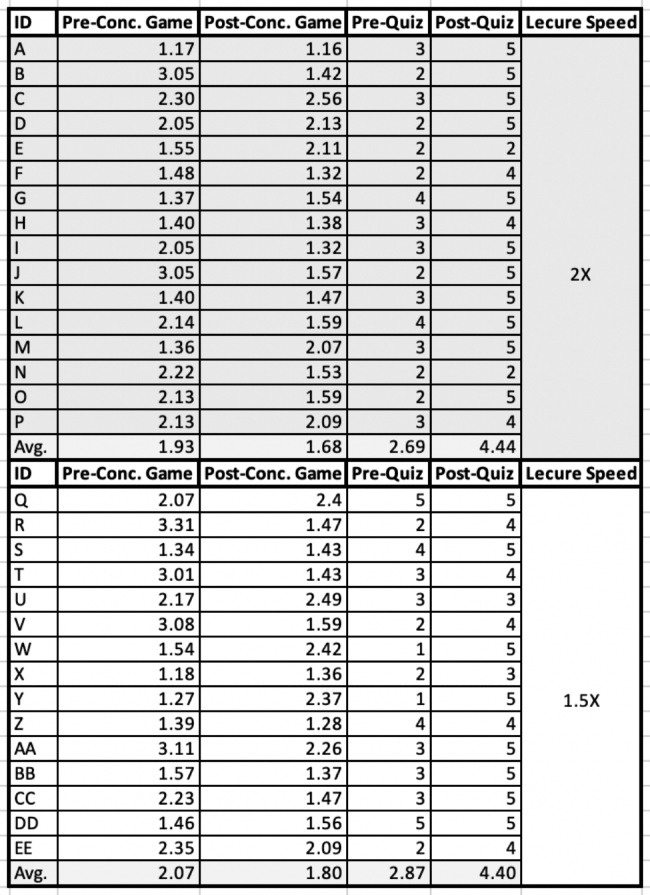
Cohort 1 Results

In Cohort 1, 16 students watched the lecture at 2x speed, and 15 students watched the lecture at 1.5x speed. In the 2x speed group, the average score of the pre-lecture concentration game was 1.93, whereas the post-lecture concentration game average was 1.68; this expressed a difference of -0.25. For this group, the pre-lecture quiz average was 2.69, whereas the post-lecture quiz average was 4.44; this expressed a difference of + 1.75. In the 1.5x speed group, the average score of the pre-lecture concentration game was 2.07, whereas the post-lecture concentration game average was 1.80; this expressed a difference of -0.27. For this group, the pre-lecture quiz average was 2.87, whereas the post-lecture quiz average was 4.40; this expressed a + 1.53 difference. This data can be seen in Fig. 4.

On average, both groups scored higher in the post-lecture quiz and took less time to complete the post-lecture concentration game. The 1.5x speed group took longer to complete the pre-lecture concentration game speed compared to the 2x speed group.

**Fig. 5:**
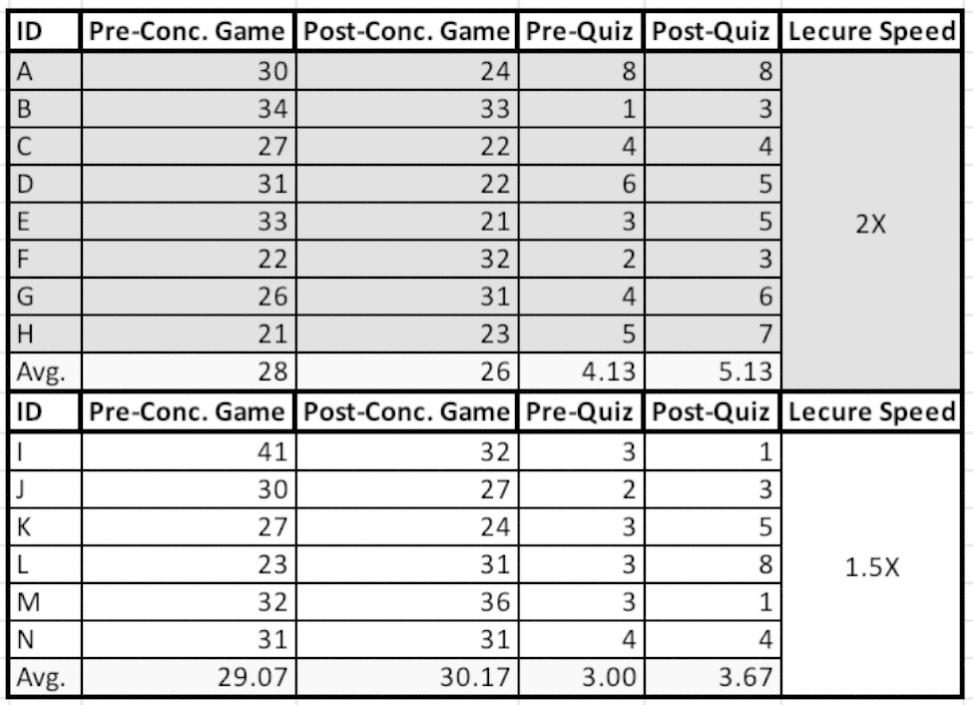
Cohort 2 Results

In Cohort 2, 8 students watched the lecture at 2x speed, and 6 students watched the lecture at 1.5x speed. In the 2x speed group, the average score of the pre-lecture concentration game was 28, whereas the post-lecture concentration game average was 26; this expressed a difference of -2. For this group, the pre-lecture quiz average was 4.13, whereas the post-lecture quiz average was 5.13; this expressed a difference of + 1. In the 1.5x speed group, the average score of the pre-lecture concentration game was 29.07, whereas the post-lecture concentration game average was 30.17; this expressed a difference of + 1.1. For this group, the pre-lecture quiz average was 3, whereas the post-lecture quiz average was 3.67; this expressed a + 0.67 difference. This data can be seen in Fig. 5.

On average, both groups scored higher in the post-lecture quiz, with negligible time difference in completion between the two groups. The 2x speed group in this cohort showed a decrease in the number of turns needed to complete the post-lecture quiz and had higher pre-lecture and post-lecture quiz scores than the 1.5x speed group.

In Cohort 3, 9 students watched the lecture at 2x speed, and 12 students watched the lecture at 1.5x speed. In the 2x speed group, the pregame average was 31.89, whereas the post-game average was 33. This expressed a difference of + 1.11. In the 1.5x speed group, the pre-game average was 31.08, while the post-game average was 32.25. This represents a difference of + 1.17.

**Fig. 6:**
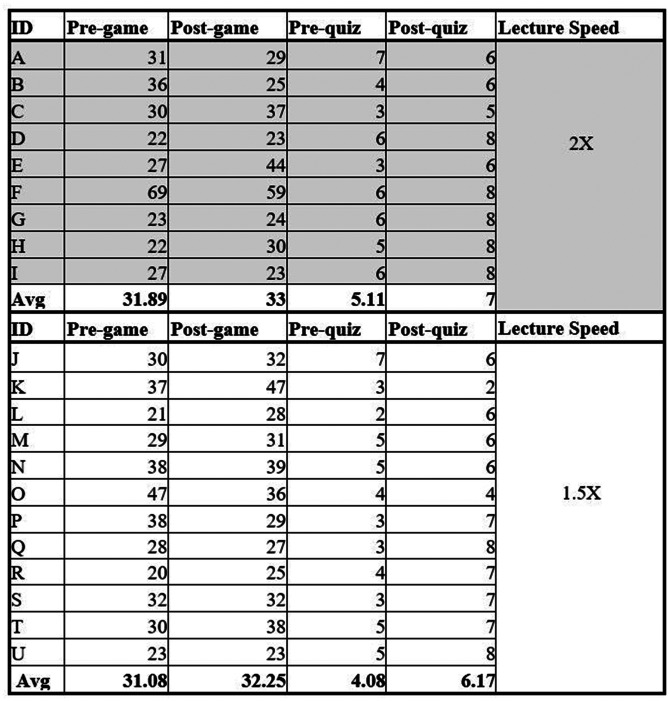
Cohort 3 Results

In the 2x speed group, the pre-lecture quiz average was 5.11, whereas the post-lecture quiz average was 7; this expressed a difference of + 1.89. In the 1.5x speed group, the pre-lecture quiz average was 4.08, whereas the post-lecture quiz was 6.17; this expressed a difference of + 2.09. This data can be seen in Fig. 6.

On average, 2x and 1.5x speed groups required one more matching attempt from the pre- to post-game. Both groups scored higher in the post-lecture quiz, with the 2x speed group scoring higher than the 1.5x speed group.

**Table 1:** t-Test: Two-Sample Assuming Equal Variances for 1st study

pre-game 2x vs. 1.5x				post-game 2x vs. 1.5x		
	*2x*	*Variable 1.5x*			*2x*	*Variable 1.5x*
Mean	1.978666667	2.072		Mean	1.678125	1.799333
Variance	0.31388381	0.569089		Variance	0.152242917	0.220235
Observations	16	15		Observations	16	15
df	26			df	27	
t Stat	-0.384688184			t Stat	-0.779196418	
P(T < = t) one-tail	0.35179859			P(T < = t) one-tail	0.221320244	
t Critical one-tail	1.70561792			t Critical one-tail	1.703288446	
P(T < = t) two-tail	0.703597181			P(T < = t) two-tail	0.442640488	
t Critical two-tail	2.055529439			t Critical two-tail	2.051830516	
**pre-test 2x vs. 1.5x**				**post-test 2x vs. 1.5x**		
	*2x*	*Variable 1.5x*			*2x*	*Variable 1.5x*
Mean	2.6875	2.866667		Mean	4.4375	4.4
Variance	0.495833333	1.552381		Variance	1.0625	0.542857
Observations	16	15		Observations	16	15
df	22			df	27	
t Stat	-0.488568772			t Stat	0.117075094	
P(T < = t) one-tail	0.314990863			P(T < = t) one-tail	0.453833545	
t Critical one-tail	1.717144374			t Critical one-tail	1.703288446	
P(T < = t) two-tail	0.629981726			P(T < = t) two-tail	0.90766709	
t Critical two-tail	2.073873068			t Critical two-tail	2.051830516	

When comparing the 1.5x and 2x speed in the first cohort of students, it was found that none of the observed differences between the pre- and post-quizzes and games were statistically significant at the conventional alpha level of 0.05. This data is observed in Table 1

The pre-game analysis showed no statistically significant difference between the 2x and 1.5x game completion speed (t(26) = -0.384688184, p = 0.704). Similarly, there was no statistically significant difference in the post-game analysis in the 2x and 1.5x game completion speed (t(27) = -0.779196418, p = 0.443).

The pretest analysis showed no statistically significant difference in test scores between the 2x and 1.5x conditions (t(22) = -0.488568772, p = 0.630). Lastly, the posttest analysis showed no statistically significant difference in the test score between 2x and 1.5x conditions (t(27) = 0.117075094, p = 0.908).

**Table 2:** t-Test: Two-Sample Assuming Equal Variances for 2nd and 3rd study combined

pre-game 2x vs. 1.5x				post-game 2x vs. 1.5x		
	*2x*	*1.5x*			*2x*	*1.5x*
Mean (matching attempts)	30.05882353	30.94444		Mean (matching attempts)	29.52941176	31.55556
Variance	121.8088235	51.34967		Variance	96.88970588	35.9085
Observations	17	18		Observations	17	18
Df	27			df	26	
t Stat	-0.279806427			t Stat	-0.730441427	
P(T < = t) one-tail	0.390879247			P(T < = t) one-tail	0.235825777	
t Critical one-tail	1.703288446			t Critical one-tail	1.70561792	
P(T < = t) two-tail	0.781758494			P(T < = t) two-tail	0.471651554	
t Critical two-tail	2.051830516			t Critical two-tail	2.055529439	
**pre-test 2x vs. 1.5x**				**post-test 2x vs. 1.5x**		
	*2x*	*1.5x*			*2x*	*1.5x*
Mean	4.647058824	3.722222		Mean	6.117647059	5.333333
Variance	3.492647059	1.624183		Variance	3.110294118	5.411765
Observations	17	18		Observations	17	18
Df	28			df	32	
t Stat	1.700796708			t Stat	1.127823783	
P(T < = t) one-tail	0.050031757			P(T < = t) one-tail	0.133889939	
t Critical one-tail	1.701130934			t Critical one-tail	1.693888748	
P(T < = t) two-tail	0.100063514			P(T < = t) two-tail	0.267779878	
t Critical two-tail	2.048407142			t Critical two-tail	2.036933343	

When comparing the 1.5x and 2x speeds in the combined second and third cohort of students, it was found that none of the observed differences between the pre- and post-quizzes and games were statistically significant at the conventional alpha level of 0.05. This data is observed in Table 2

In the pre-game analysis, there was no statistically significant difference in the mean matching attempts between the 2x and 1.5x conditions (t(27) = -0.28, p = 0.781). Similarly, in the postgame analysis, there was no statistically significant difference in the mean matching attempts between the 2x and 1.5x conditions (t(26) = -0.73, p = 0.472).

In the pretest analysis, there was no statistically significant difference in the test scores between the 2x and 1.5x conditions (t(28) = 1.70, p = 0.100). Lastly, the post-test analysis showed no statistically significant difference in the test scores between the 2x and 1.5x conditions (t(32) = 1.13, p = 0.268).

## Discussion

As the world has changed due to Covid-19, medical students not only have the stress of an ever-increasing workload but also students need to prepare for a world that continues to be affected by a pandemic. As shown in the study by Hill et al., students felt their most significant stressors were time constraints, lack of balance, and workload [1]. This study was conducted before the pandemic, and since then, Murphy et al. conducted a study at the University of California, Los Angeles (UCLA) with 108 undergraduate students who looked at asynchronous lectures, watching them at 1x, 1.5x, and 2x playback speeds [10]. As discussed in this paper, there has been conflicting efficacy in the studies done as the concept of video learning in compressed time was written about as early as 1995 in a study by Gutenko [11]. The missing link between the studies done before 2020 and now is that the Covid-19 pandemic forced the world into an online classroom format. Before the pandemic, most medical education had mandatory, in-person lecture attendance. Hence, the possibility of watching the lecture at a faster speed or the freedom to pause the lecture at any point was not an option. This makes the work conducted in the study even more crucial as there is a small pool of research to assess the pandemic’s effect on learning. Through navigating this unfamiliar territory, certain initial expectations were disproven as the study progressed. The initial expectations were that (1) memory retention and concentration may be negatively and positively influenced by lecture playback speed, and (2) comprehension of lecture content is more likely to be diminished with lecture playback speeds over 1x.

Based on this study outcome, it was found that lecture playback speed did not negatively impact memory and concentration. It is fair to note that this unexpected finding could be due to the multiple variables given, such as the supplemental visual aids, lecture slides, and practice questions. The work of this study opens the door to expand the research, refine endpoints, and assist in future medical school curriculum planning. The question then becomes how much information a medical student can be expected to learn, synthesize, and then extrapolate under timed conditions in the form of exams and boards. Mo et al. have a recent paper discussing Cognitive Load Theory and playback speed [12]. The theory is based on the concept that humans have an intrinsic cognitive load, which considers the knowledge base, expertise, and experience, versus an extrinsic cognitive load, which is based on the school’s faculty, curriculum, and expectations [12]. Though the student has some control over intrinsic cognitive load, the study learned that students found the greatest satisfaction at playback speeds of 1.5x [12]. This information can aid medical schools in trying to keep pace with the changes in a post-pandemic world and how students learn effectively. Perhaps increasing the playback speed can increase the student’s cognitive load, creating another further study option.

The results of the cohorts in this study suggest that increasing the playback speed was not deleterious to outcomes. Participants showed a significant increase in knowledge base between the pre- and post-lecture quizzes with less time spent in the actual lecture. These findings have implications beyond just academic results, as with the amount of information taught to medical students increasing consistently, students need more time for self-care and wellness. Finding ways to support students successfully through their medical school careers holistically includes the amount of time devoted to lectures. The study suggests that faster playback speeds can achieve positive results without sacrificing achievement while simultaneously allowing students more time to process and integrate other aspects of essential needs into rigorous years as a student.

### Recruitment challenges

Due to the Covid-19 Pandemic, the team could not engage students in-person or on campus, and thus, recruitment for the study could only be done online. Additionally, recent studies have shown evidence of increased email phishing in the healthcare industry [13]. As a result, there has been a heightened awareness of phishing emails at the authors’ medical institution around recruitment. Faculty, staff, and students became more cognizant of phishing schemes via institutional training programs. Consequently, students hesitated to click the links in the research email invitations. The team attempted to circumvent this by sending the email through the Student Government Association (SGA) to reduce the likelihood of the invitation being seen as a phishing attempt. Secondly, it was challenging to recruit one large group preferably, and instead had to conduct multiple small groups over time; this was again attributed to only being able to engage students via email. The team believes that in-person student outreach and “word of mouth” between students in an in-person setting would have allowed for a preferable recruitment size in one study.

### Presenter’s significance

Multiple rationales exist for deciding which speed(s) a learner may use to playback a lecture. The presenter of the lecture plays an integral role as often, the presenter’s accent may be difficult to follow. Hence, students tend to listen at the normal 1x speed and pause the lecture to fill in the gaps in understanding. Another issue of significance is the speed at which the presenter naturally speaks. Thus, for the slower-speaking presenters, the learners embrace the ability to speed up the lecture playback. For the majority, watching lectures at faster speeds can be attributed to attempting to save time in the fast-paced medical school curriculum.

### Strengths

The three cohorts experienced the research activities with minimal variances between events. The overall outcomes for each cohort consistently reveal no significant differences when lecture playback speeds were 1.5x or 2.0x, and learners did not lack concentration or long-term memory concentration. These findings will be valuable for educators navigating the return from online to in-person learning. Educators may find certain students are better acclimated to online learning and, therefore, could provide online recordings to supplement their curriculum. Educators will also be able to navigate answering questions regarding if.

students can or should be listening to recorded material at faster playback speeds.

### Limitations

Due to the restrictions of the Covid-19 pandemic, having the sessions on Zoom® made the team unable to monitor if participants were looking at other materials or paying attention while listening to the lectures. The participants were also required to complete pre- and post-lecture tests, which could not be monitored. Another limitation to consider was the students’ access to medical lectures. Pre-recorded lectures for the following week became available for all students on Sundays. Firstly, although students are remarkably busy, it was not possible to control if some of the participants had already watched the lecture the team happened to choose.

Secondly, the limitations of the video lecture tool only allow students to view lectures up to 2x playback speed, and thus, this study was limited to 0.5x, 1x, 1.5x, & 2x options.

Thirdly, in the first trial, the team attempted to use the time it took to complete the concentration game as a measure of concentration between the two groups; however, a decrease in time to complete it was noticed in all participants. This may have been due to participants rapidly clicking boxes, as there was no penalty for the number of attempts. Therefore, the authors resolved that tracking the number of attempts compared to the time to complete would reduce the variable of random clicking and finding pairs. This is the rationale as to why the team chose a different game for the 2nd and 3rd studies, which measures the number of attempts to match.

Additionally, the quiz was changed from 5 to 10 questions in the pre- and post-quiz after the first study. This was done because, during the first study, the team noticed that most students finished the questions well under the 90 s per question allotted. The relative ease of the questions also raised concerns as they were 1st and 2nd -order questions, which are more manageable than the 3rd and 4th -order-style questions present on the United States Medical Licensing Exam (USMLE), which students in the 5th semester would already be preparing to take. Lastly, each lecture has at least ten practice questions, so students usually complete ten questions after a lecture. Therefore, given these observations, the team felt the number of questions was too conservative, given their difficulty level.

Finally, students were required to submit screen images of their concentration test scores due to remote participation. Although there was no reasonable suspicion of this occurring beyond stating only attempted the concentration game once, it could not be verified whether participants attempted it multiple times.

### Future directions

Since the Covid-19 pandemic, asynchronous learning has become prevalent both out of need and convenience. Extending the research into a post-pandemic world may give helpful clues to the continued use of variable playback speed modality. Additionally, creating space for modifying issues such as supplemental aids, practice questions, and how these factors impact learning may be of value. Given that in this study, investigators selected research participation from second-year medical students only; future studies may obtain a more diverse group of medical students by including both novices first-year medical students and experienced second-year medical students. Future research could investigate how lecture playback speeds impact first-year medical school learners while comparing second-year medical students’ memory, concentration, and retention. This may help to determine whether the outcome and ultimate recommendation on using different playback speeds would be the same for both cohorts of students. Both cohorts must endure the rigors of foundational science lectures before advancing into the clinical years. Looking at this perspective from a longitudinal study may yield long-term clarity in the adaptive environment of increasing online learning resources.

This study model can also be replicated with outside study resources that can increase video speed above 2x. Beyond this speed, the authorial team believes this would be the point at which concentration and long-term memory retention would begin to decrease.

Finally, repeating the same study design but with multiple lectures, quizzes, and games may give more definitive and real-life applications of lecture playback speed on concentration and memory. Additional survey questions, “How many lectures do you watch in a row” and “How long of a break do you take between lectures” would also help validate the notion that students watch lectures in succession.

## Conclusions

Medical students use the increase in lecture playback speed as an effective tool to overcome the time needed to watch the mass volume of lecture recordings. This study revealed no significant difference in concentration or long-term memory retention when playback speed is at 1.5x versus 2x speed. Furthermore, the content comprehension of lectures was not negatively impacted as expected. Given these findings, it is recommended that students may listen to lectures using lecture playback speed at 1.5x to 2x speed without a decline in concentration or memory of content.

## Data Availability

The datasets used and/or analyzed during the current study are available from the corresponding author upon reasonable request.
